# TGF-β1 Protection against Aβ_1–42_-Induced Neuroinflammation and Neurodegeneration in Rats

**DOI:** 10.3390/ijms151222092

**Published:** 2014-12-01

**Authors:** Wei-Xing Shen, Jia-Hui Chen, Jian-Hua Lu, Yu-Ping Peng, Yi-Hua Qiu

**Affiliations:** 1 School of Biological & Basic Medical Sciences, Soochow University, 199 Renai Road, Suzhou 215123, China; E-Mail: swx@ntu.edu.cn; 2 Department of Physiology, School of Medicine, Nantong University, 19 Qixiu Road, Nantong 226001, China; E-Mails: liuzhan8301@163.com (J.-H.C.); ljhua@ntu.edu.cn (J.-H.L.); 3 Co-innovation Center of Neuroregeneration, Nantong University, 19 Qixiu Road, Nantong 226001, China

**Keywords:** TGF-β1, Alzheimer’s disease, Aβ_1–42_, T-lymphocytes, microglia

## Abstract

Transforming growth factor (TGF)-β1, a cytokine that can be expressed in the brain, is a key regulator of the brain’s responses to injury and inflammation. Alzheimer’s disease (AD), the most common neurodegenerative disorder, involves inflammatory processes in the brain in addition to the hallmarks, amyloid-β (Aβ) plaques and neurofibrillary tangles. Recently, we have shown that T-helper (Th) 17 cells, a subpopulation of CD4^+^ T-cells with high proinflammation, also participate in the brain inflammatory process of AD. However, it is poorly known whether TGF-β1 ameliorates the lymphocyte-mediated neuroinflammation and, thereby, alleviates neurodegeneration in AD. Herein, we administered TGF-β1 via the intracerebroventricle (ICV) in AD model rats, by Aβ_1–42_ injection in both sides of the hippocampus, to show the neuroprotection of TGF-β1. The TGF-β1 administration after the Aβ_1–42_ injection ameliorated cognitive deficit and neuronal loss and apoptosis, reduced amyloid precursor protein (APP) expression, elevated protein phosphatase (PP)2A expression, attenuated glial activation and alleviated the imbalance of the pro-inflammatory/anti-inflammatory responses of T-lymphocytes, compared to the Aβ_1–42_ injection alone. These findings demonstrate that TGF-β1 provides protection against AD neurodegeneration and suggest that the TGF-β1 neuroprotection is implemented by the alleviation of glial and T-cell-mediated neuroinflammation.

## 1. Introduction

Transforming growth factor (TGF)-β1, a member of three known mammalian TGF-β isoforms (TGF-β1, 2 and 3), is a pleiotropic cytokine that regulates the proliferation, differentiation and survival of various cell types [1] and also regulates the brain’s responses to injury and inflammation [2]. In the brain, TGF-β1 is highly expressed in the cerebral cortex, hippocampus, central amygdaloid nucleus, medial preoptic area, substantia nigra and brainstem [3]. Cell types expressing TGF-β1 in the brain include all glial cells, astrocytes, oligodendrocytes and microglia [1,4,5]. Neurons do not express or express TGF-β1 less in normal states [4,5,6,7,8]. However, TGF-β1 expression is largely up-regulated in perifocal neurons and reactive astroglial cells of ischemia [9], in hypertrophic astrocytes of multiple sclerosis lesions [4], in glial cells and neurons of necrotizing human brain lesions [7] and in autopsied brains of individuals who died with central nervous system (CNS) diseases [6]. These findings suggest that TGF-β1 is actively involved in various cell responses to brain injury, inflammation and diseases.

Alzheimer’s disease (AD), the most common dementia, is pathologically characterized by cerebral accumulation of amyloid-β (Aβ) peptide into Aβ plaques, neurofibrillary tangles, neuronal injury and neuroinflammation. In AD, TGF-β1 has been detected in Aβ plaques [10], and higher TGF-β1 levels were found in the cerebrospinal fluid (CSF) [11,12] and serum [13] of AD cases than in nondemented or healthy controls. Furthermore, cortical TGF-β1 mRNA levels correlate positively with the degree of cerebrovascular amyloid deposition in AD cases, and TGF-β1 immunoreactivity in these cerebral amyloid angiopathy cases is elevated along cerebral blood vessels [14] and in perivascular astrocytes [15]. In addition, in cortical brain regions of TgCRND8 mice, a mouse model of familial Alzheimer’s disease, TGF-β1 is markedly upregulated [16]. These findings strongly show that TGF-β1 has been implicated in the pathogenesis of AD.

Nevertheless, the role of TGF-β1 in AD neuropathology is controversial. In support of TGF-β1 neuroprotection, Wyss-Coray *et al.* [2] have reported that an increase in astroglial TGF-β1 production in aged transgenic mice expressing the human beta-amyloid precursor protein (hAPP) results in a reduction in parenchymal amyloid plaques and in overall Aβ load in the hippocampus and neocortex. In addition, a lack of TGF-β1 expression in neonatal *Tgfb1*(−/−) mice results in a widespread increase in degenerating neurons accompanied by a prominent microgliosis [17]. However, TGF-β1 may amplify Aβ_1–42_-mediated neurodegeneration in AD [16] and potentiate Aβ production in human astrocytes [18]. Thus, further study is needed to elucidate the role of TGF-β1 in AD. We hypothesized that TGF-β1 prominently exerts anti-inflammatory properties and, therefore, is neuroprotective in AD. Our recent work has shown that neuroinflammation in AD is also mediated by T-lymphocytes that infiltrate into brain parenchyma [19]. Herein, we focused on the anti-inflammatory property of TGF-β1 by determination of microglial and T-cell functions to show its neuroprotective mechanism in AD. In addition, TGF-β1 administration after Aβ_1–42_ invasion in this study provides a therapeutic, rather than preventive, approach to AD. This is helpful for designing a therapeutic strategy for clinical patients with AD.

## 2. Results

### 2.1. TGF-β1 Ameliorates Aβ_1–42_-Induced Impairments of Cognition and Neurons

**Figure 1:**
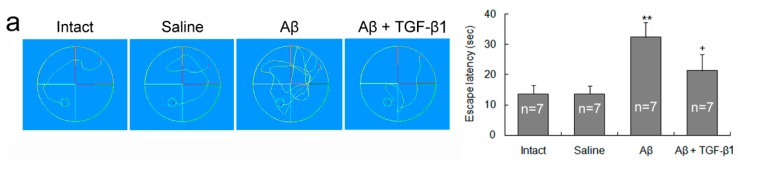

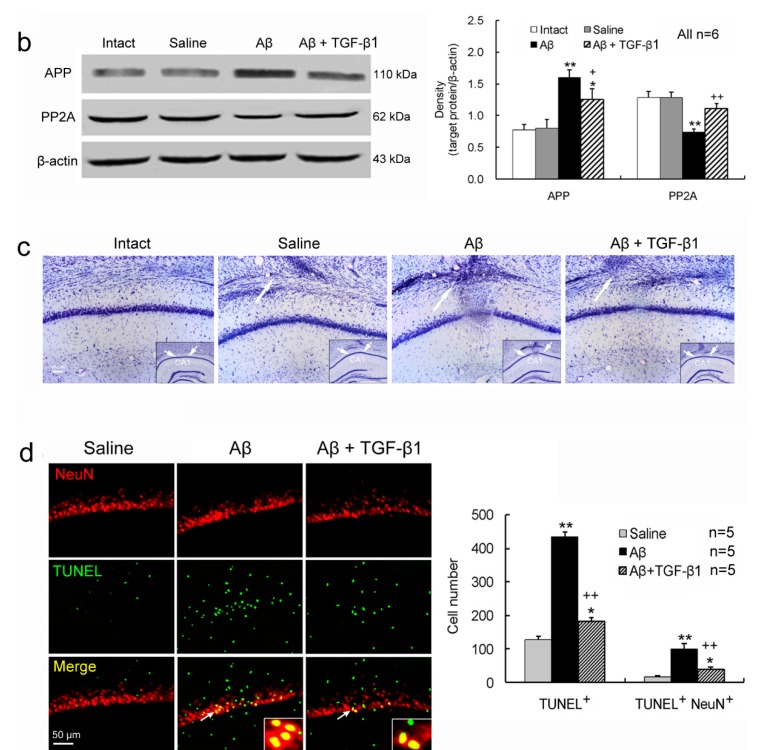
TGF-β1 treatment ameliorates Aβ_1–42_-induced deficits in cognition and neuropathology. TGF-β1 (50 ng/5 μL) was given via the intracerebroventricle (ICV) seven days following Aβ_1–42_ injection into the bilateral hippocampus of rats. On Day 3 after the TGF-β1 administration, the parameters were measured. (**a**) Escape latency in Morris water maze. (**Left**) Swimming tracks of rats in the Morris water maze. The escape latency was recorded from the beginning of entering the water for the rats (the red points) to the arrival on the platforms (the green small rounds); (**Right**) A statistical histogram of the repeated experiments; (**b**) Expression levels of APP and PP2A in the hippocampus. (**Left**) representative electrophoretic bands; (**Right**) statistics of the repeated experiments; (**c**) Nissl staining of the hippocampal CA1 region. The big arrows point at the locations where injection needles were placed and reactive gliosis is seen. Note that Aβ_1–42_ induced an obvious neuronal loss in the CA1 region and that TGF-β1 reduced this loss, reflected by the Nissl bodies. The insets within the images are general views of the hippocampus, where CA1 regions are denoted; (**d**) NeuN and TUNEL labeling around the CA1 region of the hippocampus. (**Left**) A representative image of the immunofluorescent histochemistry. The TUNEL/NeuN double-stained cells are pointed at by the arrows and magnified in the insets; (**Right**) A statistical histogram. The data are obtained by counting 15 visual fields in three hippocampal sections for each rat * *p* < 0.05, ** *p* < 0.01, *versus* intact or saline treatment; + *p* < 0.05, ++ *p* < 0.01, *versus* Aβ_1–42_ injection alone.

TGF-β1 (50 ng/5 μL) was injected in the left lateral cerebral ventricle on the seventh day after Aβ_1–42_ infusion into bilateral hippocampus of rats. The Aβ_1–42_ injection alone induced an increase in escape latency in a Morris water maze (Figure 1a), upregulation of amyloid precursor protein (APP) expression, down-regulation of protein phosphatase (PP)2A expression in the hippocampus (Figure 1b), a loss of neurons (Figure 1c) and an increase in TUNEL-stained and TUNEL/NeuN double-stained cells in the CA1 region of the hippocampus (Figure 1d), when compared with intact or saline controls. The TGF-β1 administration via the intracerebroventricle (ICV) after Aβ_1–42_ invasion significantly decreased the escape latency (Figure 1a), APP expression (Figure 1b), neuronal loss (Figure 1c) and apoptosis (Figure 1d), but elevated the PP2A expression (Figure 1b), with respect to Aβ_1–42_ injection alone.

In addition to the cognitive and pathological impairments, the Aβ_1–42_ injection also induced clinical manifestations, including reduced attention, weak activeness and depressed exploration behavior. The TGF-β1 administration improved these clinical manifestations induced by Aβ_1–42_.

### 2.2. TGF-β1 Reduces the Up-Regulation of Inflammatory Mediators and Down-Regulation of Neurotrophic Factors Induced by Aβ_1–42_ in the Hypothalamus

**Figure 2:**
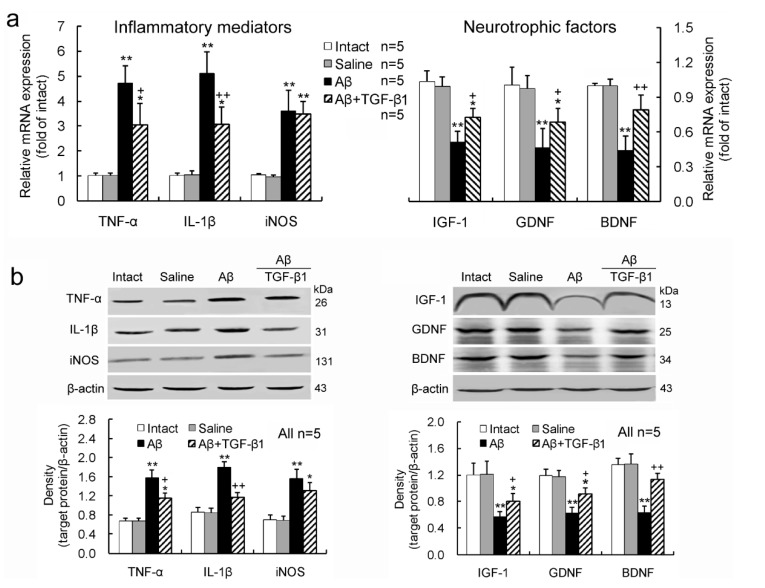
TGF-β1 reduces Aβ_1–42_-induced upregulation of inflammatory mediators and down-regulation of neurotrophic factors in the hypothalamus. TGF-β1 (50 ng/5 μL) was administered via ICV seven days after Aβ_1–42_ injection, and on Day 3 following the TGF-β1 administration, the expression levels of mRNAs (**a**) and proteins (**b**) for the inflammatory mediators and neurotrophic factors in the hippocampus were determined. * *p* < 0.05, ** *p <* 0.01, *versus* intact or saline injection; + *p* < 0.05, ++ *p* < 0.01, *versus* Aβ_1–42_ treatment alone.

The Aβ_1–42_ injection in bilateral hippocampus up-regulated mRNA (Figure 2a) and protein (Figure 2b) expression levels of tumor necrosis factor (TNF)-α, interleukin (IL)-1β and inducible nitric oxide synthase (iNOS), the inflammatory mediators, and down-regulated the expression levels of insulin-like growth factor (IGF)-1, glial-derived neurotrophic factor (GDNF) and brain-derived neurotrophic factor (BDNF), the neurotrophic factors, in the hippocampus in comparison with intact or saline controls (Figure 2a,b). TGF-β1 (50 ng/5 μL) administration via ICV reduced the upregulated TNF-α and IL-1β expression and the down-regulated IGF-1, GDNF and BDNF expression induced by Aβ_1–42_, but it did not significantly alter Aβ_1–42_-induced up-regulation of iNOS expression (Figure 2a,b).

### 2.3. TGF-β1 Alleviates Pro-Inflammatory Enhancement and Anti-Inflammatory Attenuation of T-Lymphocytes Induced by Aβ_1–42_ in the Hippocampus

**Figure 3:**
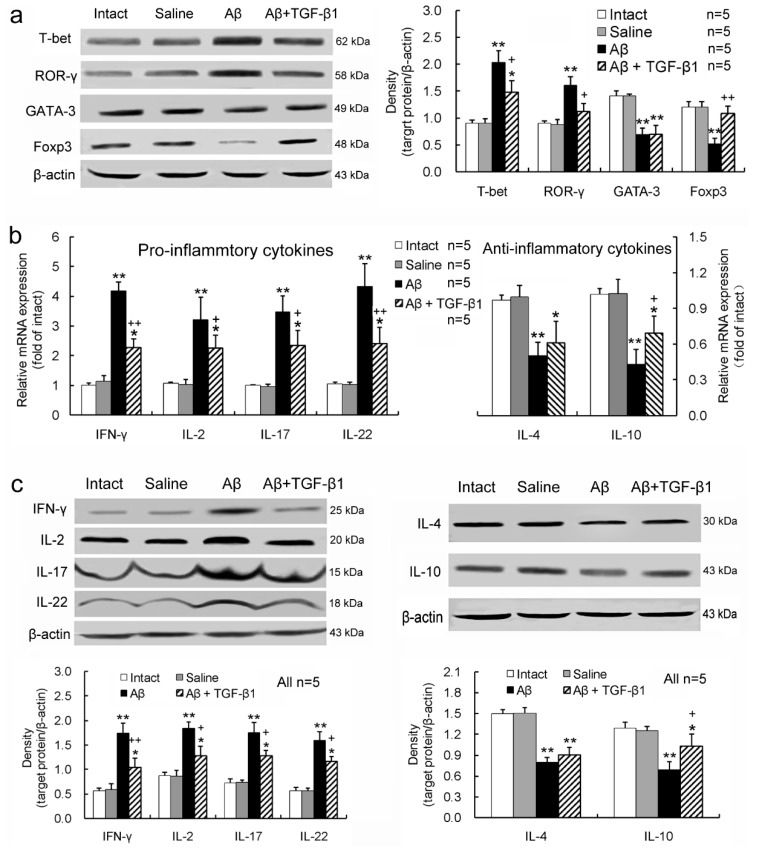
TGF-β1 alleviates pro-inflammatory enhancement and anti-inflammatory attenuation of T-lymphocytes induced by Aβ_1–42_ in the hippocampus. (**a**) Expression levels of transcription factors T-bet, ROR-γ, GATA-3 and Foxp3 specific for Th1, Th17, Th2 and Treg cells, respectively; (**b**) Gene expression levels of pro-inflammatory and anti-inflammatory cytokines in the hippocampus; (**c**) Protein expression levels of the pro-inflammatory and anti-inflammatory cytokines in the hippocampus. * *p <* 0.05, ** *p <* 0.01, *versus* intact or saline treatment; + *p <* 0.05, ++ *p <* 0.01, *versus* Aβ_1–42_ injection alone.

Expression of T-bet and ROR-γ, the specific transcription factors of helper T-(Th) 1 and Th17 cells, respectively, was upregulated, whereas the expression of GATA-3 and Foxp3, the specific transcription factors of Th2 and regulatory T-(Treg) cells, respectively, was downregulated in the hippocampus by the Aβ_1–42_ injection, compared with those of intact or saline controls (Figure 3a). TGF-β1 treatment alleviated the Aβ_1–42_-induced upregulation of T-bet and ROR-γ and downregulation of Foxp3, but it did not significantly alter the downregulated GATA-3 by Aβ_1–42_ (Figure 3a). In addition, the pro-inflammatory cytokines, interferon (IFN)-γ, IL-2, IL-17 and IL-22, were up-regulated, while the anti-inflammatory cytokines, IL-4 and IL-10, were down-regulated at the gene and protein expression levels in Aβ_1–42_-injected hippocampus (Figure 3b,c). Significantly, the TGF-β1 administration ameliorated the up-regulated pro-inflammatory cytokine expression and the down-regulated anti-inflammatory cytokine expression in the Aβ_1–42_-injected hippocampus, although it did not significantly elevate the anti-inflammatory IL-4 expression level (Figure 3b,c).

### 2.4. TGF-β1 Reverses the Aβ_1–42_-Induced Elevation of Pro-Inflammatory Cytokines and Reduction of Anti-Inflammatory Cytokine in Serum or CSF

**Figure 4:**
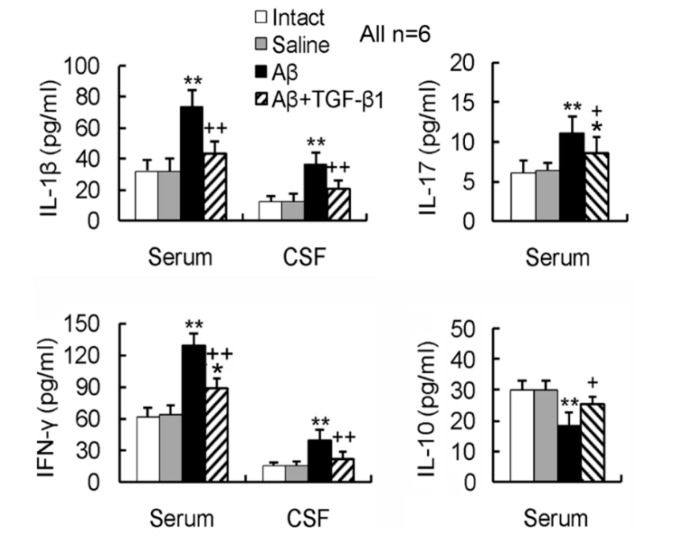
TGF-β1 reverses the Aβ_1–42_-induced elevation of pro-inflammatory cytokines and the reduction of anti-inflammatory cytokine in serum or CSF. Concentrations of the cytokines, including pro-inflammatory IL-1β, IFN-γ and IL-17 and anti-inflammatory IL-10 in serum or CSF, were examined by ELISA. * *p <* 0.05, ** *p <* 0.01, *versus* intact or saline controls; + *p <* 0.05, ++ *p <* 0.01, *versus* Aβ_1–42_ injection alone.

The pro-inflammatory cytokines, IL-1β, IFN-γ and IL-17, were elevated, while the anti-inflammatory cytokine, IL-10, was reduced in serum or CSF by the Aβ_1–42_ injection in the hippocampus (Figure 4). Notably, the elevation of pro-inflammatory cytokine concentrations and the reduction of anti-inflammatory cytokine in serum or CSF by Aβ_1–42_ invasion were basically reversed by the TGF-β1 administration via ICV (Figure 4).

## 3. Discussion

In the brain, Aβ is formed from a larger protein, named APP, via breakdown by enzymes and deposited in extracellular plaques known as senile plaques [20,21]. The formation and deposition of Aβ is an important cause for neuronal death in vulnerable regions, such as the hippocampus and neocortex, which induces the behavioral and functional deficits of AD [22]. Injection of Aβ into the hippocampus can induce neurodegenerative changes, particularly in the CA1 area, and, therefore, imitates both the pathological and behavioral characteristics of AD [23]. Although transgenic animal models for AD have been extensively used, intrahippocampal injection of Aβ_1–42_ in rat brain has been suggested as an animal model, which emphasizes the inflammatory reactivity present in the human AD brain [24]. Therefore, the present results showing the delayed escape latency in the Morris water maze, up-regulated APP and increased neuronal loss and apoptosis in the hippocampus by the Aβ_1–42_ injection demonstrate that AD-associated neurodegeneration is induced. Importantly, TGF-β1 ICV administration after Aβ_1–42_ invasion alleviated the AD-associated neurodegeneration. This suggests that TGF-β1 has neuroprotection against AD behavior and pathology. As a support to our current results, Wyss-Coray *et al.* [2] have reported that TGF-β1 is an important modifier of amyloid deposition *in vivo*. TGF-β1 also plays a pivotal role in maintaining neuronal integrity and the survival of CNS neurons of AD [17]. Thus, our present findings that exogenously added TGF-β1 ameliorated Aβ_1–42_-induced cognitive and pathological deficits provide more evidence for the neuroprotection of TGF-β1 against AD.

Although growing evidence suggests a neuroprotective role for TGF-β1 against Aβ toxicity, both for *in vitro* and *in vivo* models of AD, some reports suggest that TGF-β1 may increase Aβ accumulation in AD models [16,18,25]. We speculate that the reasons for the inconsistent effects of TGF-β1 are complicated and may include three aspects. First, TGF-β1 is a pleiotropic cytokine that influences the brain’s responses to inflammation and injury, depending on whether glial cells exist in the environment of inflammation and injury. Wyss-Coray *et al.* [2] have shown that the reduction of parenchymal plaques in hAPP/TGF-β1 transgenic mice is associated with a strong activation of microglia and an increase in inflammatory mediators. In microglial cell cultures, recombinant TGF-β1 stimulates Aβ clearance, indicating that TGF-β1 may promote microglial processes that inhibit the accumulation of Aβ in the brain parenchyma [2]. These findings suggest that glial cells are crucial targets for TGF-β1 action. Second, the effects of TGF-β1 on glial cells depend on TGF-β1 signaling. Inflammatory cytokines and Aβ induce the activation of glial cells, leading to both protective and deleterious changes that are relevant for the pathogenesis of AD. Astrocytes down-regulate microglial cell cytotoxic activation through secretion of TGF-β1, and the TGF-β1 modifies Aβ removal through the modulation of microglia [26]. However, inflammatory activation of microglia is increased and Aβ clearance is reduced in AD patients, regardless of the fact that TGF-β1 is increased in their nervous system. Tichauer and von Bernhardi [26] propose that changes in TGF-β Smad signal transduction could modify the regulation mediated by TGF-β1. They indicate that the modulation of microglial cell activation by TGF-β1, leading to increased clearance of Aβ and reduced cytotoxicity, is at least partially mediated by the Smad pathway. In addition, some evidence has shown that an impairment of the TGF-β1 signaling pathway is specific to the AD brain and, particularly, to the early phase of the disease [27,28,29]. The deficiency of TGF-β1 signaling is associated with Aβ pathology and neurofibrillary tangle formation in AD animal models. Reduced TGF-β1 signaling seems to contribute both to microglial activation and to ectopic cell-cycle re-activation in neurons, two events that contribute to neurodegeneration in the AD brain [28]. The neuroprotective features of TGF-β1 indicate the advantage of rescuing TGF-β1 signaling as a means to slow down the neurodegenerative process in AD [27,28]. Third, TGF-β1 levels may also determine its effects. Because individual TGF-β1 expression levels in the brain vary considerably between humans, the effects of TGF-β1 could have important implications for susceptibility to neurodegeneration [17].

The inflammatory mediators, TNF-α, IL-1β and iNOS, and the neurotrophic factors, IGF-1, GDNF and BDNF, are principally produced by glial cells in the brain. Therefore, the results showing that Aβ_1–42_ induces upregulation of the inflammatory mediators and down-regulation of the neurotrophic factors suggest an excessive activation of glial cells. Chronic neuroinflammation mediated by activation of glial cells has been proposed as a driving force for AD. Although the effect of TGF-β1 on Aβ deposition is controversial, the action of its inhibiting glial activation is consistently suggested. In rat primary glial cultures, TGF-β1 pretreatment reduces the production of inflammatory mediators induced by Aβ_42_ [30]. In BV-2 microglial cultures, TGF-β1 attenuates Aβ-induced microglial clustering and migration toward Aβ aggregates [31]. These studies *in vitro* directly demonstrate that TGF-β1 suppresses microglial activation. Our present results support this fact and provide further evidence *in vivo*, suggesting that TGF-β1 reduction of Aβ_1–42_-induced upregulation of inflammatory mediators and down-regulation of neurotrophic factors may be a mechanism underlying its neuroprotection in AD.

CD4^+^ T-cells, on activation and expansion, develop into four subpopulations, Th1, Th17, Th2 and Treg cells, with different cytokine profiles and distinct effector functions. Th1 and Th17 cells mainly contribute to inflammation by producing pro-inflammatory cytokines, such as IFN-γ, IL-2, IL-17 and IL-22. Th2 cells are anti-inflammatory, and Treg cells are characterized by the control of immune responses and suppression of inflammatory immune cells via producing anti-inflammatory cytokines, such as IL-4, IL-10 and TGF-β. In this study, the up-regulated T-bet and ROR-γ and the down-regulated GATA-3 and Foxp3 in the Aβ_1–42_-injected hippocampus suggest a differentiation bias of CD4^+^ T-cells towards pro-inflammatory Th1 and Th17 cells away from anti-inflammatory Th2 and Treg cells in the AD brain. In support of these results, we observed that Th1- and Th17-related pro-inflammatory cytokines were up-regulated and Th2- and Treg-related anti-inflammatory cytokines were down-regulated in the Aβ_1–42_-injected hippocampus, suggesting a functional imbalance in pro-inflammatory Th1 and Th17 cells and anti-inflammatory Th2 and Treg cells. These findings show that neuroinflammation caused by T-cell differentiation and functional imbalance is present in the PD brain. Since the blood–brain barrier (BBB) exists, the brain generally is considered as a site with lymphocyte privilege. However, over recent decades, studies have presented that the brain, particularly in AD, has lymphocyte infiltration. Recently, we have shown that Th17 cells infiltrate into brain parenchyma through disrupted the BBB and, therefore, participate in neuroinflammation and neurodegeneration in Aβ_1–42_-induced AD model rats [19]. Reports from other laboratories support the findings. In the brains of transgenic mice that overexpress APP and presenilin 1 (APP/PS1), a significant infiltration of T-cells that secrete IFN-γ or IL-17 is found [32]. Intracerebral Aβ interaction with the Aβ receptor at the BBB causes circulating T-cell infiltration in the brain in AD [33]. Furthermore, after Aβ-specific Th1 cells generated by immunization with Aβ are adoptively transferred to APP/PS1 transgenic mice, microglial activation and Aβ deposition increase, and these changes are associated with impaired cognitive function [32]. Accordingly, we infer that Th1 or Th17 cells that infiltrate into brain parenchyma in AD exacerbate neuroinflammation by facilitating of microglial activation, and as a result, neurodegeneration is aggravated. Importantly, TGF-β1 attenuated the enhancement of Th1- and Th17-pro-inflammatory responses induced by Aβ_1–42_ in the current study. In addition, TGF-β1 also elevated anti-inflammatory responses in Aβ_1–42_-injected hippocampus. Therefore, TGF-β1 can promote the balance of pro-inflammatory/anti-inflammatory responses and ameliorate neuroinflammation. This may be a critical mechanism by which TGF-β1 implements neuroprotection against AD neurodegeneration. These results provide new insight into the understanding of TGF-β1 neuroprotection by inhibiting T-cell-mediated inflammation in AD.

In addition to cytokine expression changes in the brain of AD model rats, cytokine secretion in the serum and CSF of the AD rats was also altered in this study. These results further confirm an imbalance in pro-inflammatory/anti-inflammatory responses in AD. It has been reported that patients with AD have a significant increase in serum IL-1β levels [34,35]. Our present results support and extend the evidence. Significantly, the abnormal pro-inflammatory and anti-inflammatory cytokine levels in the serum and CSF were reversed by TGF-β1 ICV administration. Although the mechanism underlying the inhibition of peripheral inflammatory response by the TGF-β1 ICV administration needs clarification, this study provides a feasible approach for the assessment of the treatment effect of AD patients by measurement of pro-inflammatory and anti-inflammatory cytokine levels in the serum.

## 4. Experimental Section

### 4.1. Animals

Four-month old Sprague-Dawley rats (Center of Experimental Animals, Nantong University, Nantong, China) were kept on a 12-h light/dark cycle and housed individually with free access to food and water. The rats were randomly divided into four groups: intact, saline injection in bilateral hippocampus, Aβ_1–42_ injection in bilateral hippocampus and TGF-β1 administration via ICV after the Aβ_1–42_ injection. The intact and saline-injected animals were used as controls. There were 12 rats for each group, and therefore, a total of 48 rats was used in the current study. All experimental procedures involving animals were carried out in accordance with the policy guidelines of the National Institutes of Health Guide for the Care and Use of Laboratory Animals (NIH Publications No. 80-23), revised 1996, and approved by the Ethical Committee of Nantong University (No. 20120316-002, 16 March 2012).

### 4.2. Preparation of Aβ_1–42_-Induced AD Rat Model

Before injected, Aβ_1–42_ (Sigma–Aldrich, St. Louis, MO, USA) was incubated in sterile saline at 37 °C for 7 days to allow the change in the assembly state of the peptide with ensuing toxicity [36]. The incubated Aβ_1–42_ solutions generally contain both fibril-like structures and different-sized oligomers [37]. Rats that had been deeply anesthetized with pentobarbital (55 mg/kg, i.p.) and mounted in a stereotactic frame (David Kopf 902-A, Tujunga, CA, USA) were injected by pressure with the incubated Aβ_1–42_ solution into each side of the hippocampus with a volume of 1 μL containing 4 μg Aβ_1–42_ using the following stereotaxic coordinates: 3.6 mm posterior to the bregma, 2.4 mm left/right to the midline and 2.8 mm ventral to the bregma [38]. The injection was performed within 5 min, and following the injection, the needle remained in the target location for 10 min to avoid Aβ_1–42_ reflux along the needle tract. After the surgery, each rat was injected with penicillin (100,000 U) in hindquarter muscle to prevent infection.

### 4.3. TGF-β1 Treatment

The TGF-β1 administration via ICV is in reference to [39] with a modification. TGF-β1 (R&D Systems, Wiesbaden, Germany) was dissolved in sterile saline in a concentration of 10 ng/μL. The TGF-β1 solution of 5 μL was injected into the left lateral cerebral ventricle seven days after Aβ_1–42_ injection with the following coordinates: 0.8 mm posterior to the bregma, 1.5 mm lateral from the midline and 3.8 mm ventral from the skull. On the third day following TGF-β1 administration, all of the measurements described below were performed.

### 4.4. Morris Water Maze

The Morris water maze (Xin Ruan XR-XM101, Shanghai, China), a circular black swim tank (160 cm in diameter and 50 cm in depth) with a small round escape platform (8 cm in diameter) within it, was filled with warm water (23 ± 1 °C) to a depth of 27 cm, and the escape platform was submerged 1 cm below the surface of the water. Before obtaining the escape latency of rats in the Morris water maze, the rats were given four trials (an alternation of a 60-s swim and a 30-s rest) per day for two consecutive days to find the hidden platform. The swimming activity of the rats was monitored by a video camera mounted overhead and automatically recorded by a video tracking system. The readout was the latency to find the hidden platform.

### 4.5. Nissl Staining

Rats were perfused with 4% paraformaldehyde (pH 7.4) after being anesthetized. The 30 μm-thick coronal sections of the brains were cut in a cryostat (Leica CM 1900-1-1, Wetzlar, Germany) after the brains were post-fixed in the same fixative for 2–4 h at 4 °C. To ensure that hippocampal sections were matched between groups, anatomical landmarks provided by the brain atlas were used. The sections were mounted on polylysine-coated slides, dried overnight, rehydrated in distilled water and then submerged in 1% cresyl violet (Sigma–Aldrich) for about 20 min until the desired depth of staining was achieved. After being rinsed in distilled water and dehydrated in graded serried ethanol, the sections were immersed in xylene, mounted in neutral balsam and then coverslipped. Nissl-positive cells in the pyramidal layer of the medial CA1 region were observed for neuronal loss.

### 4.6. TUNEL Staining

The hippocampus was coronally cut into 40 μm-thick sections. The sections, the locations of which were consistent between groups according to anatomical landmarks provided by the brain atlas, were collected and blocked with 0.3% Triton X-100 and 3% goat serum (Invitrogen, Carlsbad, CA, USA) in 0.01 M PBS (pH 7.3) for 30 min. The slices were incubated with the primary antibody to NeuN produced in rabbit (Millipore, Billerica, MA, USA), diluted at 1:200 in 0.01 M PBS, overnight at room temperature. The sections were then washed in 0.01 M PBS and incubated with the secondary antibody, Alexa Fluor 594 conjugated goat anti-rabbit IgG (Cell signaling Technology, Beverly, MA, USA), diluted in 0.01 M PBS (1:200), for 4 h at room temperature. Terminal deoxynucleotidyl transferase (TdT)-mediated deoxyuridine triphosphate (dUTP)-biotin nick end labeling (TUNEL) staining was performed using the *In Situ* Cell Death Detection Kit (Roche Applied Science, Penzberg, Germany). Briefly, after NeuN staining, the mounted coronal sections were rinsed with PBS and treated with 1% Triton-100 in PBS for 2 min on ice. The sections were rinsed in PBS and incubated for 60 min at 37 °C with 50 μL of TUNEL reaction mixture. The negative control sections were incubated for 60 min at 37 °C with 50 μL of parallel solution without TdT-buffer and biotinylated dUTP. After washing with PBS, the slices were analyzed with fluorescence microscopy. For each rat, a total of 15 visual fields around the CA1 area in three hippocampal sections was counted for the TUNEL-stained cells.

### 4.7. Western Blot Analysis

The hippocampus was homogenized in an SDS sample buffer that contained a mixture of proteinase inhibitors (Sigma–Aldrich), and then, the supernatant was collected by centrifuging at 4 °C at 12,000 rpm for 15 min. The supernatants were mixed with loading buffer, which was boiled for 10 min. The proteins were separated by 10% sodium dodecyl sulfate-polyacrylamide gel electrophoresis and transferred to a polyvinylidene difluoride membrane (Pall, Port Washington, NY, USA) using an electroblotting apparatus (Bio-Rad, Hercules, CA, USA). The membranes were blocked for 1 h at room temperature in Tris-buffered saline containing 0.1% Tween-20 and 5% dry milk and were then incubated with the primary antibodies to APP (1:1000, Millipore), PP2A (1:1000, Cell Signaling Technology), TNF-α (1:300, Abcam, Cambridge, UK), IL-1β (1:200, Santa Cruz Biotechnology, Inc., Santa Cruz, CA, USA), iNOS (1:50, Abcam), IGF-1 (1:100, Santa Cruz Biotechnology, Inc.), GDNF (1:100, Abcam), BDNF (1:100, Santa Cruz Biotechnology, Inc.), T-bet (1:100, Santa Cruz Biotechnology, Inc.), IFN-γ (1:200, Santa Cruz Biotechnology, Inc.), IL-2 (1:100, Santa Cruz Biotechnology, Inc.), ROR-γ (1:200, Santa Cruz Biotechnology, Inc.), IL-17 (1:100, Santa Cruz Biotechnology, Inc.), IL-22 (1:100, Santa Cruz Biotechnology, Inc.), GATA-3 (1:100, Santa Cruz Biotechnology, Inc.), IL-4 (1:500, R&D Systems, Wiesbaden, Germany), Foxp3 (1:200, Santa Cruz Biotechnology, Inc.) or IL-10 (1:200, Santa Cruz Biotechnology, Inc.). The membranes were probed at 4 °C overnight and incubated with IRDye 800-conjugated goat anti-rabbit IgG (1:5000, Rockland Immunochemicals, Inc., Gilbertsville, PA, USA), IRDye 800-conjugated goat anti-mouse IgG (1:5000, Rockland Immunochemicals, Inc.) or IRDye 800-conjugated donkey anti-goat IgG (1:5000, LI-COR Inc., Lincoln, NE, USA) for 1 h at room temperature, followed by visualization by an Odyssey laser scanning system (LI-COR Inc.). Blots were re-probed with monoclonal mouse anti-β-actin antibody (1:5000, Sigma–Aldrich) and reacted with IRDye 800-conjugated goat anti-mouse IgG (1:5000, Rockland Immunochemicals, Inc.) to confirm equal protein loading. The molecular weight and relative quantity of the protein bands were determined by an image analysis system (Odyssey 3.0 software, LI-COR Inc.).

### 4.8. Real-Time PCR Analysis

Total RNA of the hippocampus was extracted with TRIzol reagent (Invitrogen) according to the manufacturer’s instructions. Potentially contaminating residual genomic DNA was eliminated with RNAse-free DNAse (Promega, Madison, WI, USA). After the RNA content was determined by spectrophotometric analysis at 260 nm, 2 μg of total RNA were reversely transcribed in a 20-μL reaction used for cDNA synthesis with murine myelomonocytic lymphoma virus reverse transcriptase (Promega). Real-time quantitative PCR was performed on cDNA by using the Rotor-Gene 3000 Real-time Cycler with SYBR green I as the detection system (Corbett Research, Sydney, Australia). Each 20 μL of reaction mixture contained 1 μL of cDNA, 2 μL PCR buffer, 3.0 mM MgCl_2_, 0.2 mM of each dNTP, 0.2 μM of each pair of oligonucleotide primers and 1 U Taq DNA polymerase (Takara, Otsu, Japan). Reaction procedures were as follows: an initial step at 95 °C for 5 min, 40 cycles of 94 °C for 15 s, 62 °C for 20 s and 72 °C for 20 s. The data were collected using the instrument’s software (Rotor-Gene software, Version 6.0), and relative quantification was performed using the comparative threshold method after determining the comparative threshold values for reference (*β-actin*) and target genes (*TNF-α*, *IL-1β*, *iNOS*, *IGF-1*, *GDNF*, *BDNF*, *IFN-γ*, *IL-2*, *IL-17*, *IL-22*, *IL-4* or *IL-10*) in each sample set, according to the 2^−ΔΔ*C*t^ method, as described by the manufacturer (User Bulletin, Sydney, Australia). Changes in mRNA expression levels were calculated after normalization to *β-actin*, a house-keeping gene. To verify the specificity of the amplification reaction, melting curve analysis was performed. The primer sequences of the target genes are listed in Table 1.

**Table 1:** Sequences of PCR primers.

Gene	Sense Primer	Antisense Primer
*TNF-α*	5'-CCACCACGCTCTTCTGTCTAC-3'	5'-ATCTGAGTGTGGGGTCTGG-3'
*IL-1β*	5'-CTTCCTTGTGCAAGTGTCTG-3'	5'-CAGGTCATTCTCATCACTGTC-3'
*iNOS*	5'-CAGCTGGGCTGTACAAACCTT-3'	5'-CATTGGAAGTGAAGCGTTTCG-3'
*IGF-1*	5'-TTTTACTTCAACAAGCCCACA-3'	5'-CATCCACAATGCCCGTCT-3'
*GDNF*	5'-ATTCAAGCCACCATCAAAAG-3'	5'-TCAGTTCCTCCTTGGTTTCG-3'
*BDNF*	5'-ATCCCATGGGTTACACGAAGGAAG-3'	5'-AGTAAGGGCCCGAACATACGATTG-3'
*IFN-γ*	5'-GCCCTCTCTGGCTGTTACTG-3'	5'-TACCGTCCTTTTGCCAGTTC-3'
*IL-2*	5'-CCATGATGCTCACGTTTAAATTTT-3'	5'-CATTTTCCAGGCACTGAAGATG-3'
*IL-17*	5'-TGGACTCTGAGCCGCAATG-3'	5'-GGCGGACAATAGAGGAAACG-3'
*IL-22*	5'-AGCGGTGATGACCAGAACA-3'	5'-CTCAGGGACATAAACAGCAGA-3'
*IL-4*	5'-ACCTTGCTGTCACCCTGTTCT-3'	5'-CTCTCTCAGAGGGCTGTCGTTA-3'
*IL-10*	5'-TGGCAACCCAAGTAACCCT-3'	5'-CACCCACTTCCCAGTCAGC-3'
*β-actin*	5'-CGTTGACATCCGTAAAGACC-3'	5'-TAGAGCCACCAATCCACAC-3'

### 4.9. Enzyme-Linked Immunosorbent Assay (ELISA)

Blood was taken from the right ventricle of rats, and the serum was collected by centrifugation at 3000 rpm for 20 min. CSF of the rats was withdrawn by foramen magnum puncture. Concentrations of target cytokines, IFN-γ, IL-1β, IL-17 and IL-10, in the serum and/or CSF were measured by ELISA kits (eBioscience, San Diego, CA, USA) according to the manufacturers’ guidelines.

### 4.10. Statistical Analysis

Data were expressed as the means ± standard deviation (M ± SD) of each group. Statistical analyses were performed with the Statistics Package for Social Science (SPSS, 12.0, SPSS, Chicago, IL, USA). The data were subjected to one-way analysis of variance (ANOVA) for comparison of the differences between the various treatments in each experiment. Following ANOVA, the Student–Newman–Keuls test was used for pairwise comparisons of the data. Differences were considered statistically significant at *p <* 0.05.

## 5. Conclusions

The Aβ_1–42_ injection in the bilateral hippocampus of rats induces cognitive impairment, APP upregulation and neuronal loss and apoptosis in the hippocampus. These changes are accompanied by neuroinflammation mediated by glial cells and T-cells. TGF-β1 administration via ICV after the Aβ_1–42_ injection ameliorates the behavioral and pathological impairments. These effects of TGF-β1 are associated with its attenuation of inflammatory responses caused by the activation of glial cells and by the imbalance of pro-inflammatory/anti-inflammatory T-cells. Thus, TGF-β1, as an effective anti-inflammatory cytokine, may be used to decrease the risk of AD development in new therapeutic strategies.
